# Chemoprevention of Lung Cancer with a Combination of Mitochondria-Targeted Compounds

**DOI:** 10.3390/cancers14102538

**Published:** 2022-05-21

**Authors:** Qi Zhang, Donghai Xiong, Jing Pan, Yian Wang, Micael Hardy, Balaraman Kalyanaraman, Ming You

**Affiliations:** 1 Center for Cancer Prevention, Houston Methodist Research Institute, Houston, TX 77030, USA; qzhang@houstonmethodist.org (Q.Z.); dxiong@houstonmethodist.org (D.X.); jpan@houstonmethodist.org (J.P.); ywang8817c@houstonmethodist.org (Y.W.); 2 Aix-Marseille University, CNRS, ICR, UMR 7273, 13013 Marseille, France; micael.hardy@univ-amu.fr; 3 Department of Biophysics, Medical College of Wisconsin, 8701 Watertown Plank Road, Milwaukee, WI 53226, USA; balarama@mcw.edu; 4 Center for Disease Prevention Research, Medical College of Wisconsin, 8701 Watertown Plank Road, Milwaukee, WI 53226, USA

**Keywords:** mitochondria-targeted honokiol, mitochondria-targeted lonidamine, mitochondrial bioenergetics, lung cancer, single-cell RNA sequencing

## Abstract

**Simple Summary:**

Previous reports showed that mitochondria-targeted honokiol and mitochondria-targeted lonidamine potently inhibit complex-I- and complexes-I/II-induced respiration and cancer cell proliferation. In this study, we investigated the efficacy of combining mitochondria-targeted honokiol and mitochondria-targeted lonidamine treatments for lung cancer prevention. We found that their combination exhibited striking tumor inhibition in the benzo[*a*]pyrene-induced murine lung tumor model without causing detectable side effects. Using single-cell RNA sequencing, we found combined treatment has a clear advantage in that it can significantly inhibit two oncogenic pathways—STAT3 signaling and AKT/mTOR/p70S6K signaling. Such dual inhibition may contribute to the greater efficacy of the combined drug treatment. Therefore, the combination provides a novel option for lung cancer chemoprevention.

**Abstract:**

Combined treatment targeting mitochondria may improve the efficacy of lung cancer chemoprevention. Here, mitochondria-targeted honokiol (Mito-HNK), an inhibitor of mitochondrial complex I and STAT3 phosphorylation, and mitochondria-targeted lonidamine (Mito-LND), an inhibitor of mitochondrial complexes I/II and AKT/mTOR/p70S6K signaling, were evaluated for their combinational chemopreventive efficacy on mouse lung carcinogenesis. All chemopreventive treatments began one-week post-carcinogen treatment and continued daily for 24 weeks. No evidence of toxicity (including liver toxicity) was detected by monitoring serum levels of alanine aminotransferase and aspartate aminotransferase enzymes. Mito-HNK or Mito-LND treatment alone reduced tumor load by 56% and 48%, respectively, whereas the combination of Mito-HNK and Mito-LND reduced tumor load by 83%. To understand the potential mechanism(s) of action for the observed combinatorial effects, single-cell RNA sequencing was performed using mouse tumors treated with Mito-HNK, Mito-LND, and their combination. In lung tumor cells, Mito-HNK treatment blocked the expression of genes involved in mitochondrial complex ǀ, oxidative phosphorylation, glycolysis, and STAT3 signaling. Mito-LND inhibited the expression of genes for mitochondrial complexes I/II, oxidative phosphorylation, and AKT/mTOR/p70S6K signaling in lung tumor cells. In addition to these changes, a combination of Mito-HNK with Mito-LND decreased arginine and proline metabolism, N-glycan biosynthesis, and tryptophan metabolism in lung tumor cells. Our results demonstrate that Mito-LND enhanced the antitumor efficacy of Mito-HNK, where both compounds inhibited common targets (oxidative phosphorylation) as well as unique targets for each agent (STAT3 and mTOR signaling). Therefore, the combination of Mito-HNK with Mito-LND may present an effective strategy for lung cancer chemoprevention.

## 1. Introduction

Lung cancer is the leading cause of cancer death in the United States, and cigarette smoking is the predominant cause of this disease. Former smokers remain at an elevated risk, and the number of lung cancer cases in former smokers is expected to rise. Preventing lung cancer development in at-risk populations, such as former smokers, is an important strategy to reduce lung cancer mortality [1]. Because patients receiving prevention treatment do not have overt disease, such treatments must not only be effective but also have a very low risk of side effects [2].

Honokiol (HNK), a natural compound present in magnolia bark extracts, has a favorable safety profile and has been shown to prevent development of several types of cancer in animal models [3]. HNK has been shown to inhibit ATP levels and mitochondrial respiration and increase reactive oxygen species to suppress tumor growth [3]. In previous studies, HNK was conjugated with triphenylphosphonium (TPP^+^) to become mitochondria-targeted HNK (Mito-HNK) [4]. We found that TPP^+^ attachment facilitates its mitochondrial accumulation, increasing its potency and efficacy (compared with HNK without TPP^+^ attachment) against highly metastatic lung cancer lines in vitro, and against orthotopic lung tumor xenografts and brain metastases in vivo [4].

Lonidamine (LND) in combination with standard-of-care chemotherapeutics has undergone clinical trials for multiple cancers [5,6]. LND is known to target energy metabolism primarily by inhibiting the mitochondrial pyruvate carrier, respiratory chain complexes I/II, the mitochondrial permeability transition pore, and hexokinase II [7]. Mitochondria-targeted LND (Mito-LND) was synthesized by linking LND with TPP^+^ [8]. Mito-LND was found to facilitate autophagic cell detachment resulting in better inhibition of lung cancer growth and brain metastasis than non-conjugated LND [8].

Previous reports showed that HNK and LND inhibit complex-I- and complex-II-induced respiration and proliferation in cancer cells [3,7]. We reported that Mito-HNK and Mito-LND more potently inhibit complex-I- and complexes I/II-induced respiration and cancer cell proliferation [4,8]. In this study, we investigated the efficacy of combining Mito-HNK and Mito-LND treatment for lung cancer prevention. We found that their combination exhibited striking tumor inhibition in the benzo[*a*]pyrene- (B[*a*]P) induced murine lung tumor model without causing detectable side effects. Using single-cell RNA sequencing (scRNA-seq), we found that Mito-HNK treatment decreased the expression of genes associated with complex I, oxidative phosphorylation (OXPHOS), and glycolysis, while increasing cell death pathways. Mito-LND treatment alone significantly decreased the expression of genes associated with complex I, complex II, and OXPHOS, and increased the expression of genes associated with autophagy. Similarly, Mito-LND treatment alone significantly decreased cysteine and methionine metabolism, while it hyperpolarized the mitochondrial membrane potential in lung adenoma cells. Therefore, the combination provides a novel option for lung cancer chemoprevention.

## 2. Methods

### 2.1. Reagents and Animals

**Figure 1:**
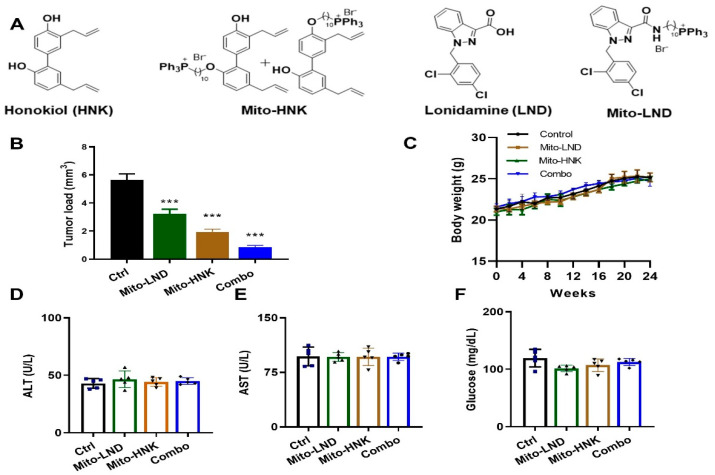
Efficacy of Mito-HNK, Mito-LND, and their combination in the B[*a*]P-induced lung cancer model. (**A**) Structures of HNK, Mito-HNK, LND, and Mito-LND. (**B**) Efficacy on tumor load. (**C**) Body weights. (**D**–**F**) Plasma levels of liver enzymes and glucose (*n* = 5). Data are presented as mean ± SEM; *** *p* < 0.001.

B[*a*]P and tricaprylin were purchased from Sigma Chemical Co. (St. Louis, MO, USA). Chromium Single Cell 3′ v3 Reagent Kits (10x Genomics) and NextSeq 500/550 High Output sequencing reagent Kits v2 (150 cycles) (Illumina, San Diego, CA, USA) were used according to the manufacturer’s protocol [9,10,11]. LKR13 cells, a mouse lung adenocarcinoma line that expresses mutant KrasG12D on the SV129 background, were a generous gift from Dr. Jonathan M. Kurie (MD Anderson Cancer Center, Houston, TX, USA). LKR13-Luc cells were generated by transfecting LKR13 cells with a lentivirus expressing firefly luciferase (PLV-10064-200; Cellomics Technology, LLC, Halethrope, MD, USA). H2030-BrM3 cells were a generous gift from Dr. Joan Massage (Memorial Sloan Kettering Cancer Center, New York, NY, USA). All cell lines were grown in RPMI (Roswell Park Memorial Institute) 1640 medium (Cat# 11875; Thermo Fisher Scientific, Wiltham, MA, USA) supplemented with 10% fetal bovine serum at 37 °C in 5% carbon dioxide. A/J mice, FVB mice, and SV129 mice were purchased from Jackson Laboratory (Bar Harbor, ME, USA). Mito-HNK and Mito-LND were synthesized as previously described [4,8] (Figure 1A).

### 2.2. Chemopreventive Efficacy of Mito-HNK on Lung Tumorigenesis

To characterize the efficacy of Mito-HNK and Mito-LND on suppressing lung carcinogenesis, the B[*a*]P-induced lung tumor model in A/J mice was used [4,8]. Six-week-old female A/J mice were injected with B[*a*]P (single i.p. dose, 100 mg/kg in 0.2 mL tricaprylin). One week after the B[*a*]P dose, mice were randomized into the following control and treatment groups: (1) control, (2) 37.5 µmol/kg body weight HNK, and (3) 3.8 µmol/kg body weight Mito-HNK. Treatments were administered by oral gavage five times per week. After 19 weeks of treatment, mice were euthanized (Figure S1). In the second experiment, mice were randomized into the following groups: (1) control, (2) 15 µmol/kg body weight Mito-LND, (3) 3.8 µmol/kg body weight Mito-HNK, and (4) combination. After 24 weeks of treatment, mice were euthanized. Lungs were fixed and evaluated under a dissecting microscope to obtain the surface tumor count and individual tumor diameter. Tumor volume was calculated based on the formula V = 4πr (3)/3. Tumor load (the total tumor volume in each mouse) was calculated from the sum of all tumors. Body weights were measured weekly. Upon termination of the experiments, serum from control and treated groups were collected and analyzed by Marshfield Labs for cholesterol, glucose, and liver function enzymes alanine transaminase (ALT), and aspartate aminotransferase (AST) [12].

### 2.3. scRNA-seq Experiment of Mice Lung Tumors

For scRNA-seq, B[*a*]P-induced lung tumors from the second experiment were harvested and pooled from different treatment groups at the end of the study, then minced and digested at 37 °C for 20 min with mouse tumor dissociation buffer (Miltenyi Biotec, Auburn, CA, USA) to generate single-cell suspensions per the manufacturer’s instructions. The lung tumors were separated from the adjacent normal tissue before being pooled, and about five tumors were pooled from each mouse for scRNA-seq. CD45 is a transmembrane protein tyrosine phosphatase located on most nucleated hematopoietic cells; CD45 is used as the marker to differentiate immune cells from other non-immune epithelial and stromal cells. Single-cell suspensions were stained with CD45 surface markers, and the CD45^−^ single cells were flow-sorted and then spun down at 300× *g* for 5 min and counted manually with a Neubauer chamber. Approximately 1.6 × 10 [4] cells were loaded onto the 10× Chromium Controller per the manufacturer’s instructions. ScRNA-seq libraries were generated by Chromium Single Cell 3′ v3 Reagent Kits (10× Genomics, Pleasanton, CA, USA) and sequenced using NextSeq 500/550 High Output sequencing reagent Kits v2 (150 cycles) (Illumina) according to the manufacturer’s protocol. There were two replicates for each of the experimental groups (control, Mito-HNK, Mito-LND, combination).

### 2.4. scRNA-seq Data Analysis

Raw sequencing data were de-multiplexed and converted to gene-barcode matrices using the Cell Ranger (version 2.2.0) mkfastq and count functions, respectively (10x Genomics). The mouse reference genome mm10 was used for alignment. Data were further analyzed in R (version 3.4.0) using Seurat (version 3) [13,14]. The number of genes detected per cell, the number of UMIs (unique molecular identifiers), and the percent of mitochondrial genes were plotted, and outliers (i.e., cells that expressed less than 200 or more than 2500 genes) were removed to filter out doublets (two single cells) and dead cells. Differences in the number of UMIs and percentage of mitochondrial reads were regressed out. Raw UMI counts were normalized and log transformed. The single cell data were aligned and projected in a two-dimensional space through t-distributed stochastic neighbor embedding or uniform manifold approximation and projection to allow identification of different cell populations using the Seurat program. For the metabolic pathway analyses, we downloaded the signature gene sets from the KEGG database (http://www.kegg.jp, accessed on 1 March 2022). When scoring cells for the expression of downloaded gene signatures, we used the AddModuleScore function implemented in Seurat. The lung tumor cells were separated from normal cells using scCancer software version 2.2.1 [15].

### 2.5. Statistical Analysis

General statistical analyses were performed using GraphPad Prism 9.0 software. One-way ANOVA were used for a column, multiple columns, and group analyses, respectively. * *p* < 0.05, ** *p* < 0.01, and *** *p* < 0.001 were considered as statistically significant.

## 3. Results

### 3.1. Efficacy of Mito-HNK in Inhibiting Lung Tumor Progression

In this study, we tested the effect of combining Mito-HNK and Mito-LND in treating B[*a*]P-induced lung tumorigenesis using two different doses of Mito-LND for the combination. Lung tumor incidence was 100% in each group. In the first experiment, A/J mice were treated with vehicle control, HNK (37.5 umol/kg), or Mito-HNK (3.8 µmol/kg body weight). Mito-HNK showed similar efficacy at a dose 10 times lower than HNK (Figure S1). In the second experiment, A/J mice were treated with vehicle control, Mito-LND (15 µmol/kg body weight), Mito-HNK (3.8 µmol/kg body weight), or a combination of Mito-HNK and Mito-LND. We showed that treatment with Mito-HNK or Mito-LND alone reduced tumor load by 66% and 43%, respectively, whereas their combination reduced the tumor load by 86% (Figure 1B). Body weights between controls and treatment groups were not significantly different (Figure 1C). We also did not observe any changes in liver enzymes or glucose levels due to treatment (Figure 1D–F) in the second experiment.

### 3.2. scRNA-seq Identified the Pathway Changes in Mouse Lung Adenoma Cells Associated with Mito-HNK Treatment

**Figure 2:**
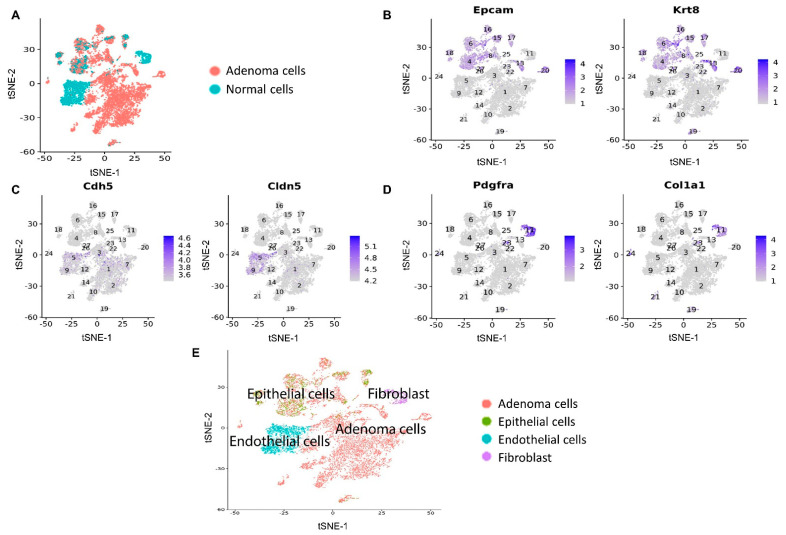
Clustering analysis of scRNA-seq data from CD45^−^ flow-sorted lung cells from control and Mito-HNK groups. (**A**) Lung adenoma cells were identified using scCancer software. Marker gene expression for (**B**) epithelial cells, (**C**) endothelial cells, and (**D**) fibroblasts. (**E**) Annotation of CD45^−^ cells into the four identified cell populations.

To identify mechanisms underlying the improved antitumor responses in mice treated with Mito-HNK, we conducted scRNA-seq analyses in replicate cell samples from both control and treatment groups. The expression of canonical markers for different types of cell populations was studied to annotate the cell clusters identified using the Seurat program. We used flow cytometry to isolate the CD45^−^ cell populations. The CD45^−^ mouse lung cells were classified into four populations: adenoma cells, epithelial cells, endothelial cells, and fibroblasts (Figure 2).

**Figure 3:**
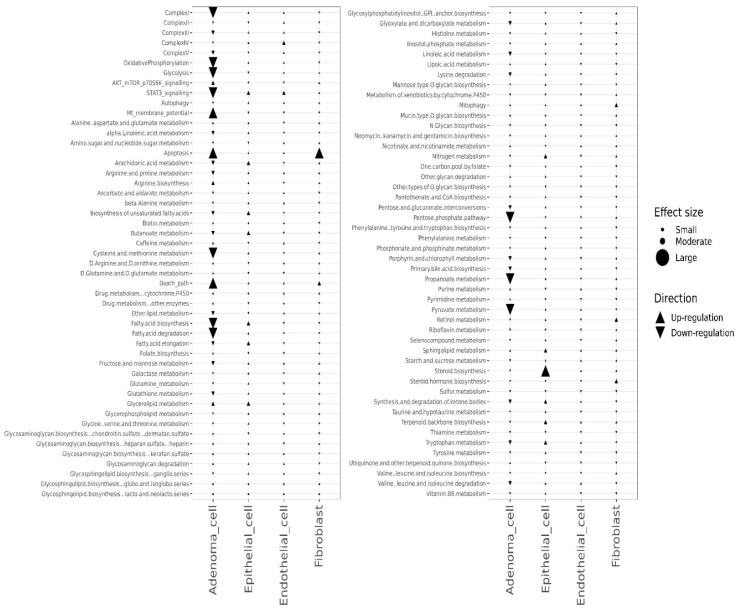
Pathway changes between control and Mito-HNK groups in the CD45^−^ cell populations. KEGG metabolism pathways and other pathways are compared between the control and Mito-HNK treated treatment groups.

**Figure 4:**
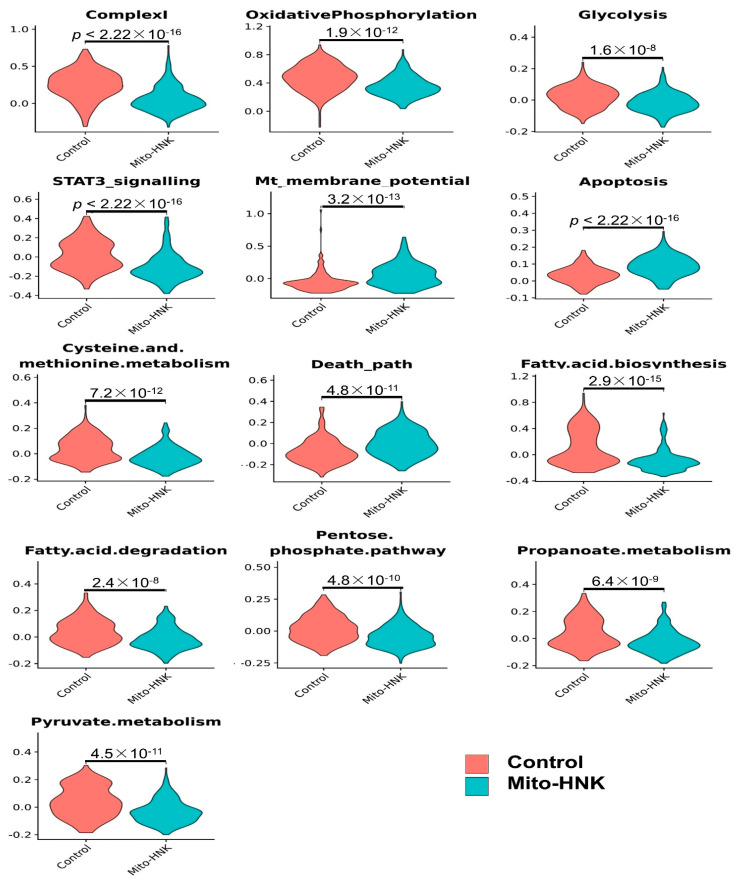
Significant pathway changes associated with Mito-HNK treatment in lung adenoma cells. In lung adenoma cells, Mito-HNK treatment decreased the expression of genes associated with complex I, OXPHOS, glycolysis, and STAT3 signaling, while the treatment increased the expression of genes associated with apoptosis and cell death pathways. In addition, Mito-HNK treatment inhibited cysteine and methionine metabolism, fatty acid metabolism, the pentose phosphate pathway, and propanoate and the pyruvate metabolism, while the treatment hyperpolarized mitochondrial membrane potential in lung adenoma cells.

We further analyzed pathway changes in the different types of CD45^−^ cells that could be associated with Mito-HNK treatment. We screened the KEGG metabolism pathways and molecular pathways underlying the antitumor efficacy of Mito-HNK treatment according to our previous findings [4]. Overall results across the CD45^−^ cell types and pathways are shown in Figure 3. We found that Mito-HNK treatment increased the expression of genes associated with steroid synthesis in normal epithelial cells and those associated with apoptosis in cancer-associated fibroblasts. No significant changes in the expression of genes associated with Mito-HNK treatment in the endothelial cells were observed (Figure 3). In lung adenoma cells, Mito-HNK treatment decreased the expression of genes associated with complex I, OXPHOS, glycolysis, and STAT3 signaling, while it increased the expression of genes associated with apoptosis and cell death pathways (Figure 3 and Figure 4). In addition, Mito-HNK treatment inhibited cysteine and methionine metabolism, fatty acid metabolism, the pentose phosphate pathway, and propanoate and pyruvate metabolism, while the treatment hyperpolarized mitochondrial membrane potential in lung adenoma cells. These newly identified metabolic pathway changes could also contribute to the efficacy of Mito-HNK in inhibiting lung adenoma cells.

### 3.3. Mito-LND Treatment Altered Multiple Pathways in Mouse Lung Adenoma Cells

**Figure 5:**
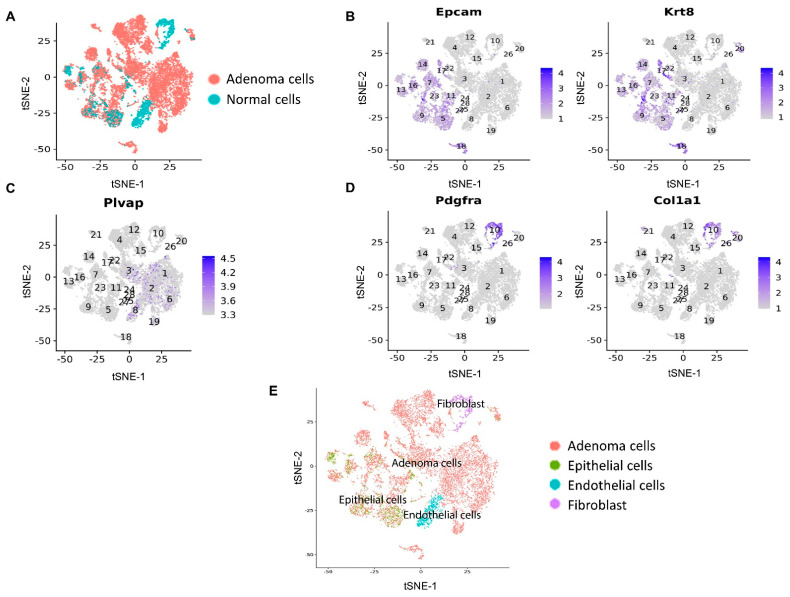
Clustering analysis based on scRNA-seq data of CD45^−^ marker flow sorted mouse lung samples from control and Mito-LND treated mice. (**A**) Lung adenoma cells were identified using scCancer software. Marker gene expression for (**B**) epithelial cells, (**C**) endothelial cells, and (**D**) fibroblasts. (**E**) Annotation of CD45^−^ cells into the four identified cell populations.

We conducted scRNA-seq analyses on single cells from the mouse lung samples of both control and Mito-LND treatment groups. The CD45^−^ mouse lung cells were classified into four populations: adenoma cells, epithelial cells, endothelial cells, and fibroblasts. The overall results across CD45^−^ cell types and pathways are shown in Figure 5.

**Figure 6:**
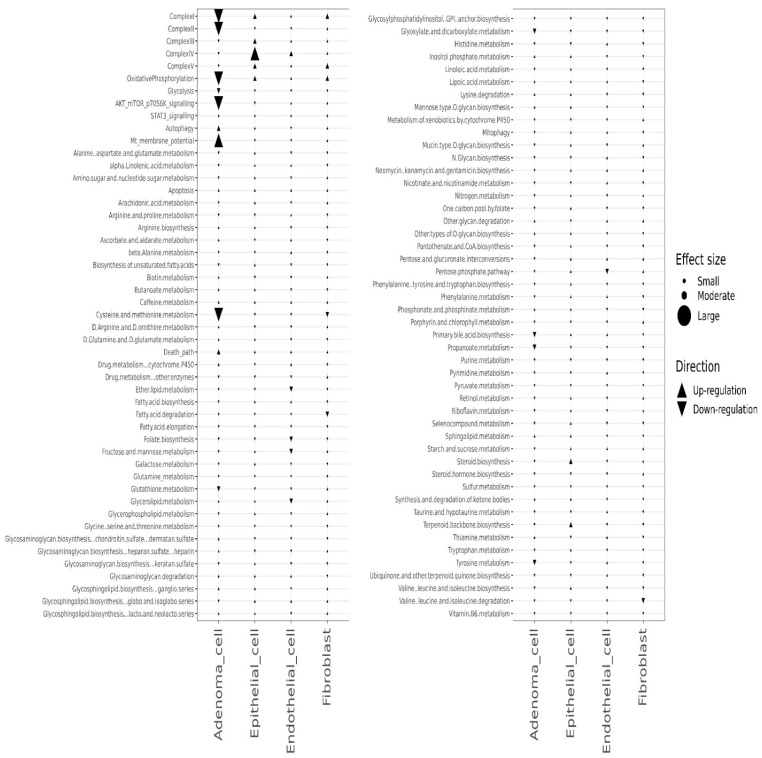
Pathway changes in the CD45^−^ cell populations from control and Mito-LND treated groups. KEGG metabolism pathways and other pathways were compared between the control and Mito-LND treated groups.

**Figure 7:**
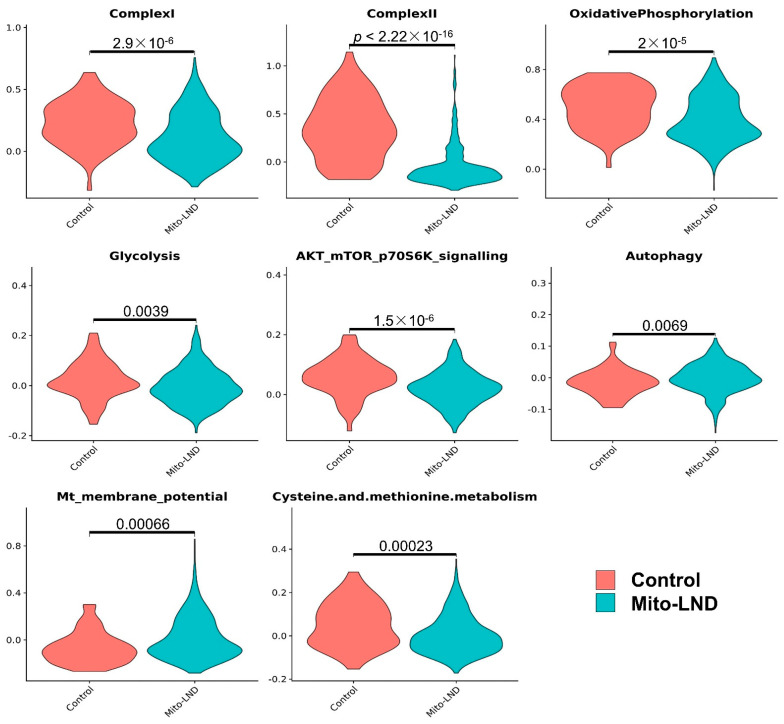
Pathway changes associated with Mito-LND treatment in lung adenoma cells. In the lung adenoma cells, Mito-LND treatment significantly decreased the expression of genes associated with complex I, complex II, OXPHOS and AKT/ mTOR/p70S6K signaling, and increased the expression of genes associated with autophagy. In addition, Mito-LND treatment significantly decreased cysteine and methionine metabolism, while treatment hyperpolarized the mitochondrial membrane potential in lung adenoma cells.

Except for the increase in the expression of genes associated with complex IV in normal epithelial cells, Mito-LND treatment had no major effect on metabolic pathways in normal CD45^−^ cells, i.e., epithelial cells, endothelial cells, and fibroblasts. In lung adenoma cells, Mito-LND treatment significantly decreased the expression of genes associated with complex I, complex II, OXPHOS, and AKT/mTOR/p70S6K signaling, and increased the expression of genes associated with autophagy (Figure 6 and Figure 7), supporting our previous results. In addition, Mito-LND significantly decreased cysteine and methionine metabolism, while hyperpolarizing mitochondrial membrane potential in lung adenoma cells (Figure 6 and Figure 7).

### 3.4. Effects of the Combined Treatment (Mito-HNK and Mito-LND) on Pathway Activities

**Figure 8:**
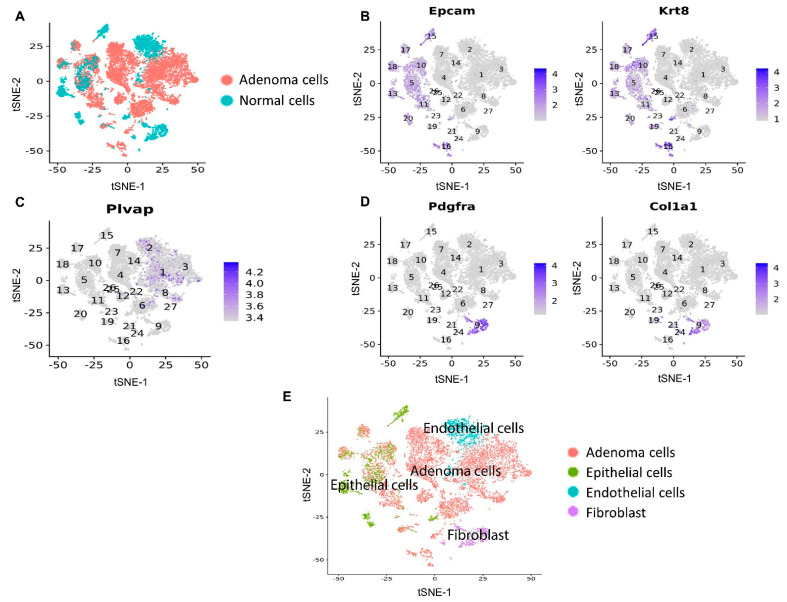
Clustering analysis based on scRNA-seq data from the CD45^−^ marker flow-sorted mouse lung samples of control and combined treatment groups. (**A**) Lung adenoma cells were identified based on the cell malignancy estimation. Marker gene expression for (**B**) epithelial cells, (**C**) endothelial cells, and (**D**) fibroblasts. (**E**) Annotation of the CD45^−^ cells into the four identified cell populations.

Similar to the Mito-HNK and Mito-LND scenarios, we identified the above-mentioned four CD45^−^ cell populations from scRNA-seq data in the combined treatment and control groups (Figure 8).

**Figure 9:**
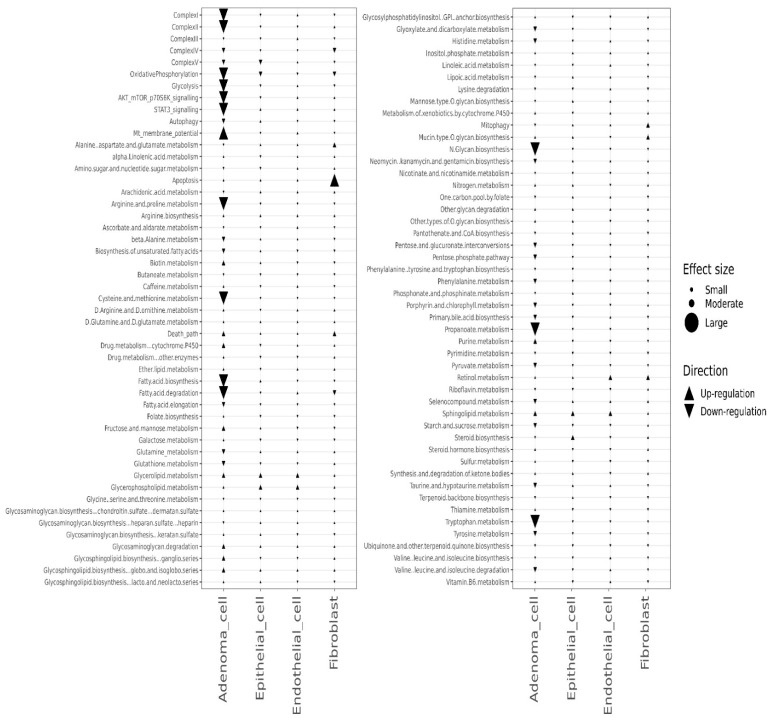
Pathway alterations in CD45^−^ cells from control vs combination treated mice. KEGG metabolism pathways and other pathways in CD45^−^ mouse lung cells were compared between the control and combined treatment groups.

**Figure 10:**
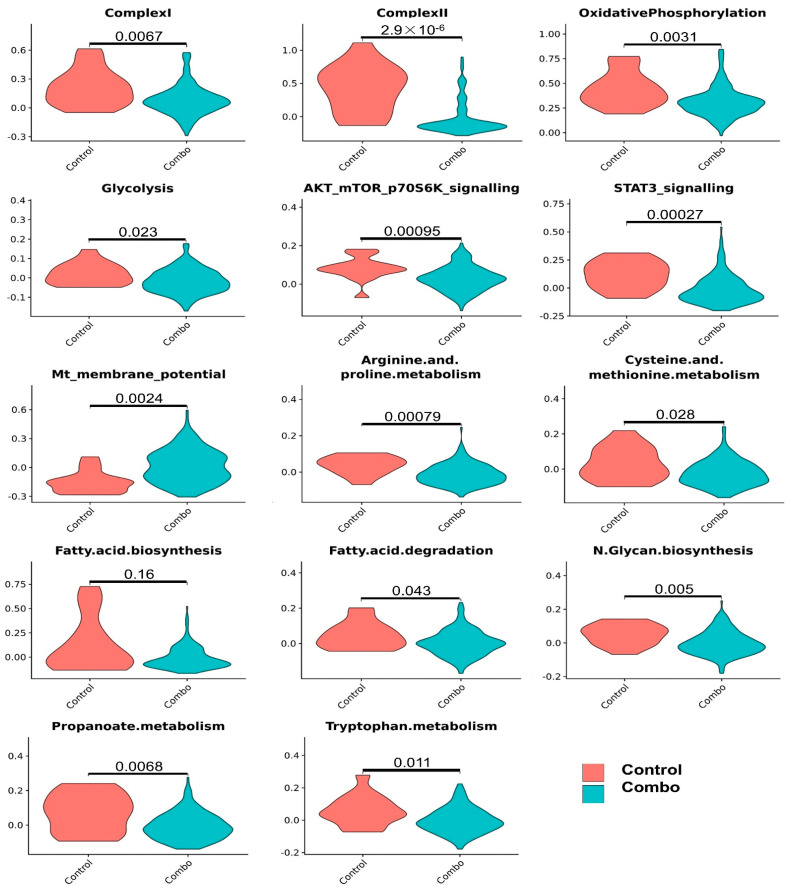
Significantly altered pathways in lung adenoma cells from mice given the combined treatment. In lung adenoma cells, the combined treatment decreased the activities of complex I, complex II, OXPHOS, glycolysis, AKT/mTOR/p70S6K signaling, STAT3 signaling, arginine and proline metabolism, cysteine and methionine metabolism, fatty acid metabolism, N-glycan biosynthesis, propanoate metabolism, and tryptophan metabolism. In contrast, combined treatment resulted in hyperpolarized mitochondrial membrane potential.

The combined treatment had no major effect on the metabolic and key signaling pathways in epithelial cells, endothelial cells, and fibroblasts. In lung adenoma cells, the combined treatment decreased the expression of genes associated with complex I, complex II, OXPHOS, glycolysis, AKT/mTOR/p70S6K signaling, STAT3 signaling, arginine and proline metabolism, cysteine and methionine metabolism, fatty acid metabolism, N-glycan biosynthesis, propanoate metabolism, and tryptophan metabolism, and it resulted in hyperpolarized mitochondrial membrane potential (Figure 9 and Figure 10). Compared with Mito-HNK or Mito-LND treatment alone, the combined treatment significantly decreased arginine and proline metabolism, N-glycan biosynthesis, and tryptophan metabolism in lung adenoma cells (Figure 10). These additional changes could reflect the complementary effects of the combined treatment on improving the overall antitumor efficacy, as observed in our animal study.

## 4. Discussion

The advantages of TPP^+^-based targeting of molecules are the stability of the TPP^+^ cation, low chemical reactivity toward cellular components, and ability to modify hydrophobicity by tethering alkylated linker side chains to various drugs [16,17]. HNK and LND were conjugated to TPP^+^ to synthesize Mito-HNK and Mito-LND with the hypothesis that they could target mitochondria more specifically. The resulting mitochondria-targeted compounds are more effective. The present study demonstrated for the first time the potent chemopreventive efficacy of Mito-HNK, Mito-LND, and a combination of the two compounds against lung tumorigenesis. After 24 weeks, treatment with the single compounds or their combination did not show any systemic toxicities, such as body weight loss, elevated liver enzymes, or altered glucose levels. Furthermore, each compound alone exhibited strong efficacy, and a combination of the two compounds showed an additive effect (Figure 1). The results of this study support the testing of these compounds in clinical trials for lung cancer prevention.

Results from the scRNA-seq analyses verified the pathway changes we previously reported for lung tumor cells. For example, in lung adenoma cells, Mito-HNK treatment inhibited mitochondrial complex I, OXPHOS, and glycolysis, and suppressed the STAT3 pathway [4]. We showed that Mito-LND treatment can inhibit mitochondrial bioenergetics, OXPHOS, and glycolysis, and can inactivate AKT/mTOR/p70S6K signaling in lung cancer cells. The scRNA-seq data identified some new pathway changes by individual compounds on lung tumor cells, including upregulation of mitochondrial membrane potential and downregulation of cysteine and methionine metabolism, fatty acid metabolism, the pentose phosphate pathway, and propanoate and pyruvate metabolism. The enhancement of mitochondrial membrane potential by both mitochondria-targeted compounds in only tumor cells suggests that a hyperpolarized tumor cell mitochondrial membrane allows for selective accumulation of TPP^+^ conjugates in tumor cell mitochondria versus normal cells [18]. This may explain the wider and stronger effects of mitochondria-targeted compounds observed in tumor cells that are lacking in normal cells. In the present study, Mito-HNK treatment inhibited the glycolytic pathway, whereas treatment with Mito-LND did not appreciably affect glycolysis. However, together, Mito-HNK and Mito-LND inhibited both glycolysis and mitochondrial complexes I/II. At present, we do not have an explanation for the differential in vivo effects of Mito-HNK and Mito-LND on glycolysis.

Interestingly, scRNA-seq results revealed that Mito-HNK and Mito-LND, alone and in combination, induced unexpected changes in essential amino acids that are required for cancer cell growth. Methionine, an essential amino acid, is not made de novo in humans and animals and can only be obtained from the diet. Methionine restriction has been shown to inhibit cancer cell growth and metabolism [19]. Methionine plays a critical role in DNA methylation in tumor suppressor genes. Tumor cells exhibit an enhanced activation of methionine recycling [20,21]. S-adenosylmethionine, the universal methyl group donor, is generated by adding ATP to methionine. Following methylation, S-adenosylmethionine is recycled back to methionine. Methionine is transported into cells by the solute carrier family (SLC). Mitochondrial carrier SLC25A26, located in the inner mitochondrial membrane, is responsible for transporting S-adenosylmethionine into mitochondria. Disruption in this transport mechanism could severely affect the methionine recycling mechanism. A novel copper complex, CTB, conjugated to a TPP+ group, inhibited methionine recycling via regulation of SLC25A26 [22,23]. Thus, it is likely that this indirect effect on methionine recycling is involved in the Mito-HNK- and Mito-LND-induced decrease in methionine in tumor cells. Emerging research indicates that decreasing methionine recycling in tumors could enhance immunotherapy [24]. Methionine is a precursor to cysteine. Both Mito-HNK and Mito-LND caused a decrease in cysteine levels. Glutathione, which is synthesized from cysteine, is minimally decreased by Mito-HNK/Mito-LND treatment. Previous research indicates that a decrease in methionine supplementation induces a compensatory increase in glutathione in tissues [19]. Fatty acid oxidation is one of the sources of energy production in cancer cells [25,26]. Fatty acids play a crucial role in cancer progression and metastasis through reprogramming and remodeling in the tumor microenvironment. Therapeutic targeting of fatty acid metabolism includes mitochondria and oncogenic signal transduction pathways (e.g., PI3-AKT-mTOR). Inhibition of mitochondrial fatty acid transporters and inhibition of mitochondrial OXPHOS significantly abrogated tumor cell growth [26]. Mito-HNK and Mito-LND potently inhibited OXPHOS genes for complex I and complex II, as shown by the RNA seq data. This suggests that mitochondrial targeting with Mito-HNK and Mito-LND may be an effective strategy to thwart fatty acid metabolism in tumors.

With combined Mito-HNK and Mito-LND treatment, we found that Mito-HNK significantly inhibited only complex I, while Mito-LND inhibited both complexes I/II, while the combined treatment significantly inhibited both complex I and complex II. Another major energy metabolism pathway, glycolysis, was also significantly inhibited by the combined treatment. Furthermore, the combined treatment resulted in additional new pathway changes that were not significantly changed by treatment with either Mito-HNK or Mito-LND alone. For example, the combined treatment significantly decreased arginine and proline metabolism, tryptophan metabolism, and N-glycan biosynthesis in lung adenoma cells; these effects were not observed in mice treated with either Mito-HNK or Mito-LND alone. Arginine and proline are nonessential amino acids that are essential for the growth of cancer cells [27,28]. Arginine signaling plays a vital role in cancer cell proliferation (1). Arginine directly activates mTOR, a nutrient signaling kinase implicated in cancer. Intracellular biosynthesis of arginine involves the reaction between citrulline and aspartate catalyzed by argininosuccinate-1 as part of the urea cycle [29]. Aspartate production is inhibited by mitochondrial respiration inhibitors [30,31,32]. Here, we found that Mito-HNK and Mito-LND inhibited complex-I- and complex-II-induced mitochondrial respiration and metabolites, including aspartate, resulting in decreased biosynthesis of arginine. Amino acid starvation strategies for cancer treatment, such as arginine and proline starvation, are in various stages of clinical application [27,28]. Tryptophan is an essential amino acid whose metabolism is the source of one-carbon units for cancer cells [33], and tryptophan metabolism is a common target in cancer and other diseases [34]. Our results suggest that combined therapy with Mito-HNK and Mito-LND inhibit the metabolism of these amino acids with better efficacy than each alone. The underlying mechanisms remain elusive, but may relate to the cross-talk between amino acid metabolism and cell signaling pathways. For example, one study showed that the suppression of mTORC1/STAT3/Notch1 prevented anomalies caused by excessive amino acids [35]. We found Mito-HNK treatment inhibited the STAT3 pathway, and Mito-LND treatment inhibited the AKT/mTOR pathway. Therefore, the combined treatment involving both compounds could inhibit both signaling pathways to suppress amino acid metabolism in lung adenoma cells. N-glycan biosynthesis is another pathway in lung adenoma that is downregulated by the combined treatment. Glycans play key roles in the biological processes of cancer, such as cancer signaling, epithelial-mesenchymal transition, cancer progression, and metastasis [36,37]. Tumor-associated glycans or glycoproteins have been used as biomarkers in cancer [31]. Disrupting N-glycan biosynthesis in tumor cells has been found to boost the efficacy of cancer therapies [38,39]. Therefore, downregulation of N-glycan biosynthesis induced by the combined treatment could boost its antitumor efficacy over that of Mito-HNK or Mito-LND treatment alone. Previous reports have suggested that glycosylation activation may require simultaneous activation of the JAK/STAT3 and PI3K/AKT pathways [40,41]. We observed little effect on glycosylation by Mito-HNK or Mito-LND treatment alone, while we found a downregulation of N-Glycan biosynthesis with the use of the combo treatment, which supports the possibility that the combined effect of Mito-HNK and Mito-LND treatment may inhibit glycosylation by inhibiting both STAT3 and AKT/mTOR signaling pathways.

## 5. Conclusions

In this study, we demonstrated that Mito-HNK is a potent inhibitor of mouse lung carcinogenesis with approximately 10× more efficacy than its parental compound, HNK. Mechanistically, we discovered that Mito-HNK primarily targets lung tumor cells and blocked the expression of genes involved in mitochondrial complex ǀ, OXPHOS, glycolysis, and STAT3 signaling. We observed that Mito-HNK alone and the combined treatment had dual inhibition effects on fatty acid metabolism and OXPHOS, while Mito-LND only inhibited OXPHOS. This was not unexpected, because fatty acids can act as uncouplers of oxidative phosphorylation in mitochondria [42]. There is no absolute correlation between fatty acid metabolism and mitochondrial respiration. Our data suggests that the dual inhibition effects, particularly the inhibition of fatty acid metabolism in the combo group, mostly depended on the component compound of Mito-HNK, rather than Mito-LND. We also show that Mito-LND can inhibit lung carcinogenesis by inhibiting the expression of genes for mitochondrial complexes I/II, OXPHOS, and AKT/mTOR/p70S6K signaling in lung tumor cells. Furthermore, the combination of Mito-HNK and Mito-LND decreased arginine and proline metabolism, N-glycan biosynthesis, and tryptophan metabolism in lung tumor cells, in addition to the effects exhibited when each compound was used alone. Our results demonstrate that Mito-LND enhances the antitumor efficacy of Mito-HNK, with both compounds inhibiting shared targets (OXPHOS and glycolysis), while simultaneously acting separately on targets unique to each agent (STAT3 and mTOR signaling pathways). Combined treatment has a clear advantage in that it can significantly inhibit two oncogenic pathways—STAT3 signaling and AKT/mTOR/p70S6K signaling. Such dual inhibition may contribute to the greater efficacy of the combined drug treatment. Finally, cytochrome P450 enzymes are the predominant enzymes responsible for the metabolic activation of carcinogens, such as the conversion of B[a]P to B[a]P diols [43]. Our scRNA-seq data (Figure 3, Figure 6 and Figure 9) showed that the cytochrome P450 pathway was not changed in either lung adenoma cells or normal cells by Mito-HNK, Mito-LND, or the combined treatment, which attests to the safety of these drugs. The low toxicity of Mito-HNK and Mito-LND, coupled with their shared ability to cross the blood–brain barrier, makes them highly attractive preventive agents against lung cancer brain metastases. Therefore, the combination of Mito-HNK with Mito-LND may represent a highly effective chemopreventive approach for mitigating lung cancer development, progression, and metastasis.

## Data Availability

The data that support the findings of this study are available from the corresponding author upon reasonable request.

## Supplementary Materials

The following supporting information can be downloaded at: https://www.mdpi.com/article/10.3390/cancers14102538/s1, Figure S1: Efficacy of HNK and Mito-HNK in the B[*a*]P-induced lung cancer model. Upper panel: experimental design; Lower pane: Efficacy on tumor load. *** *p* < 0.001.

## References

[1] Herbst R.S., Heymach J.V., Lippman S.M. (2008). Lung cancer. N. Engl. J. Med..

[2] Lippman S.M., Hawk E.T. (2009). Cancer prevention: From 1727 to milestones of the past 100 years. Cancer Res..

[3] Pan J., Zhang Q., Liu Q., Komas S.M., Kalyanaraman B., Lubet R.A., Wang Y., You M. (2014). Honokiol inhibits lung tumorigenesis through inhibition of mitochondrial function. Cancer Prev. Res..

[4] Pan J., Lee Y., Cheng G., Zielonka J., Zhang Q., Bajzikova M., Xiong D., Tsaih S.W., Hardy M., Flister M. (2018). Mitochondria-targeted honokiol confers a striking inhibitory effect on lung cancer via inhibiting complex I activity. iScience.

[5] De Lena M., Lorusso V., Latorre A., Fanizza G., Gargano G., Caporusso L., Guida M., Catino A., Crucitta E., Sambiasi D.A. (2001). Paclitaxel, cisplatin and lonidamine in advanced ovarian cancer. A phase II study. Eur. J. Cancer.

[6] Cervantes-Madrid D., Romero Y., Duenas-Gonzalez A. (2015). Reviving lonidamine and 6-diazo-5-oxo-L-norleucine to be used in combination for metabolic cancer therapy. Biomed. Res. Int..

[7] Nancolas B., Guo L., Zhou R., Nath K., Nelson D.S., Leeper D.B., Blair I.A., Glickson J.D., Halestrap A.P. (2016). The anti-tumour agent lonidamine is a potent inhibitor of the mitochondrial pyruvate carrier and plasma membrane monocarboxylate transporters. Biochem. J..

[8] Cheng G., Zhang Q., Pan J., Lee Y., Ouari O., Hardy M., Zielonka M., Myers C.R., Zielonka J., Weh K. (2019). Targeting lonidamine to mitochondria mitigates lung tumorigenesis and brain metastasis. Nat. Commun..

[9] Pan J., Chen Y., Zhang Q., Khatun A., Palen K., Xin G., Wang L., Yang C., Johnson B.D., Myers C.R. (2021). Inhibition of lung tumorigenesis by a small molecule CA170 targeting the immune checkpoint protein VISTA. Commun. Biol..

[10] Chen Y., Zander R.A., Wu X., Schauder D.M., Kasmani M.Y., Shen J., Zheng S., Burns R., Taparowsky E.J., Cui W. (2021). BATF regulates progenitor to cytolytic effector CD8(+) T cell transition during chronic viral infection. Nat. Immunol..

[11] Khatun A., Kasmani M.Y., Zander R., Schauder D.M., Snook J.P., Shen J., Wu X., Burns R., Chen Y.G., Lin C.W. (2021). Single-cell lineage mapping of a diverse virus-specific naive CD4 T cell repertoire. J. Exp. Med..

[12] Zhang Q., Pan J., Lubet R.A., Komas S.M., Kalyanaraman B., Wang Y., You M. (2015). Enhanced antitumor activity of 3-bromopyruvate in combination with rapamycin in vivo and in vitro. Cancer Prev. Res..

[13] Butler A., Hoffman P., Smibert P., Papalexi E., Satija R. (2018). Integrating single-cell transcriptomic data across different conditions, technologies, and species. Nat. Biotechnol..

[14] Satija R., Farrell J.A., Gennert D., Schier A.F., Regev A. (2015). Spatial reconstruction of single-cell gene expression data. Nat. Biotechnol..

[15] Guo W., Wang D., Wang S., Shan Y., Liu C., Gu J. (2021). scCancer: A package for automated processing of single-cell RNA-seq data in cancer. Brief. Bioinform..

[16] Zielonka J., Joseph J., Sikora A., Hardy M., Ouari O., Vasquez-Vivar J., Cheng G., Lopez M., Kalyanaraman B. (2017). Mitochondria-targeted triphenylphosphonium-based compounds: Syntheses, mechanisms of action, and therapeutic and diagnostic applications. Chem. Rev..

[17] Kalyanaraman B., Cheng G., Hardy M., Ouari O., Lopez M., Joseph J., Zielonka J., Dwinell M.B. (2018). A review of the basics of mitochondrial bioenergetics, metabolism, and related signaling pathways in cancer cells: Therapeutic targeting of tumor mitochondria with lipophilic cationic compounds. Redox. Biol..

[18] Huang M., Myers C.R., Wang Y., You M. (2021). Mitochondria as a novel target for cancer chemoprevention: Emergence of mitochondrial-targeting agents. Cancer Prev. Res..

[19] Wanders D., Hobson K., Ji X. (2020). Methionine restriction and cancer biology. Nutrients.

[20] Wang Z., Yip L.Y., Lee J.H.J., Wu Z., Chew H.Y., Chong P.K.W., Teo C.C., Ang H.Y.K., Peh K.L.E., Yuan J. (2019). Methionine is a metabolic dependency of tumor-initiating cells. Nat. Med..

[21] Newman A.C., Maddocks O.D.K. (2017). One-carbon metabolism in cancer. Br. J. Cancer.

[22] Jin C., Li Y., Su Y., Guo Z., Wang X., Wang S., Zhang F., Zhang Z., Shao J., Zheng S. (2020). Novel copper complex CTB regulates methionine cycle induced TERT hypomethylation to promote HCC cells senescence via mitochondrial SLC25A26. Cell Death Dis..

[23] Li M., Shao J., Guo Z., Jin C., Wang L., Wang F., Jia Y., Zhu Z., Zhang Z., Zhang F. (2020). Novel mitochondrion-targeting copper(II) complex induces HK2 malfunction and inhibits glycolysis via Drp1-mediating mitophagy in HCC. J. Cell. Mol. Med..

[24] Bian Y., Li W., Kremer D.M., Sajjakulnukit P., Li S., Crespo J., Nwosu Z.C., Zhang L., Czerwonka A., Pawłowska A. (2020). Cancer SLC43A2 alters T cell methionine metabolism and histone methylation. Nature.

[25] Harris A.L. (2020). Development of cancer metabolism as a therapeutic target: New pathways, patient studies, stratification and combination therapy. Br. J. Cancer.

[26] Salunkhe S., Mishra S.V., Ghorai A., Hole A., Chandrani P., Dutt A., Chilakapati M., Dutt S. (2020). Metabolic rewiring in drug resistant cells exhibit higher OXPHOS and fatty acids as preferred major source to cellular energetics. Biochim. Biophys. Acta Bioenerg..

[27] Kuo M.T., Chen H.H.W., Feun L.G., Savaraj N. (2021). Targeting the proline-glutamine-asparagine-arginine metabolic axis in amino acid starvation cancer therapy. Pharmaceuticals.

[28] Geck R.C., Toker A. (2016). Nonessential amino acid metabolism in breast cancer. Adv. Biol. Regul..

[29] Chen C.L., Hsu S.C., Ann D.K., Yen Y., Kung H.J. (2021). Arginine signaling and cancer metabolism. Cancers.

[30] Sullivan L.B., Gui D.Y., Hosios A.M., Bush L.N., Freinkman E., Vander Heiden M.G. (2015). Supporting aspartate biosynthesis is an essential function of respiration in proliferating cells. Cell.

[31] Helenius I.T., Madala H.R., Yeh J.J. (2021). An asp to strike out cancer? Therapeutic possibilities arising from aspartate’s emerging roles in cell proliferation and survival. Biomolecules.

[32] Cheng C.T., Qi Y., Wang Y.C., Chi K.K., Chung Y., Ouyang C., Chen Y.R., Oh M.E., Sheng X., Tang Y. (2018). Arginine starvation kills tumor cells through aspartate exhaustion and mitochondrial dysfunction. Commun. Biol..

[33] Newman A.C., Falcone M., Uribe A.H., Zhang T., Athineos D., Pietzke M., Vazquez A., Blyth K., Maddocks O.D.K. (2021). Immune-regulated IDO1-dependent tryptophan metabolism is source of one-carbon units for pancreatic cancer and stellate cells. Mol. Cell.

[34] Platten M., Nollen E.A.A., Rohrig U.F., Fallarino F., Opitz C.A. (2019). Tryptophan metabolism as a common therapeutic target in cancer, neurodegeneration and beyond. Nat. Rev. Drug Discov..

[35] Li H., Lee J., He C., Zou M.H., Xie Z. (2014). Suppression of the mTORC1/STAT3/Notch1 pathway by activated AMPK prevents hepatic insulin resistance induced by excess amino acids. Am. J. Physiol. Endocrinol. Metab..

[36] Silsirivanit A. (2019). Glycosylation markers in cancer. Adv. Clin. Chem..

[37] Mereiter S., Balmana M., Campos D., Gomes J., Reis C.A. (2019). Glycosylation in the era of cancer-targeted therapy: Where are we heading?. Cancer Cell.

[38] Wang Y.N., Lee H.H., Hsu J.L., Yu D., Hung M.C. (2020). The impact of PD-L1 N-linked glycosylation on cancer therapy and clinical diagnosis. J. Biomed. Sci..

[39] Greco B., Malacarne V., De Girardi F., Scotti G.M., Manfredi F., Angelino E., Sirini C., Camisa B., Falcone L., Moresco M.A. (2022). Disrupting N-glycan expression on tumor cells boosts chimeric antigen receptor T cell efficacy against solid malignancies. Sci. Transl. Med..

[40] Zegeye M.M., Lindkvist M., Fälker K., Kumawat A.K., Paramel G., Grenegård M., Sirsjö A., Ljungberg L.U. (2018). Activation of the JAK/STAT3 and PI3K/AKT pathways are crucial for IL-6 trans-signaling-mediated pro-inflammatory response in human vascular endothelial cells. Cell Commun. Signal..

[41] Riethmueller S., Somasundaram P., Ehlers J.C., Hung C.W., Flynn C.M., Lokau J., Agthe M., Düsterhöft S., Zhu Y., Grötzinger J. (2017). Proteolytic origin of the soluble human IL-6R in vivo and a decisive role of N-glycosylation. PLoS Biol..

[42] Schonfeld P., Schild L., Kunz W. (1989). Long-chain fatty acids act as protonophoric uncouplers of oxidative phosphorylation in rat liver mitochondria. Biochim. Biophys. Acta.

[43] Reed L., Arlt V.M., Phillips D.H. (2018). The role of cytochrome P450 enzymes in carcinogen activation and detoxication: An in vivo-in vitro paradox. Carcinogenesis.

