# Supplementary material for: Chemoprevention of Lung Cancer with a Combination of Mitochondria-Targeted Compounds
Source: Cancers (Basel). 2022 May 21;14(10):2538. doi: 10.3390/cancers14102538 (PMC9140024; doi:10.3390/cancers14102538)
Supplement: Supplementary file 1 [file cancers-14-02538-s001.zip › cancers-1707111-supplementary.pdf]

Supplementary Materials

Chemoprevention of Lung Cancer with a Combination of Mito-chondria-Targeted Compounds

Qi Zhang, Donghai Xiong, Jing Pan, Yian Wang, Micael Hardy, Balaraman Kalyanaraman and Ming You

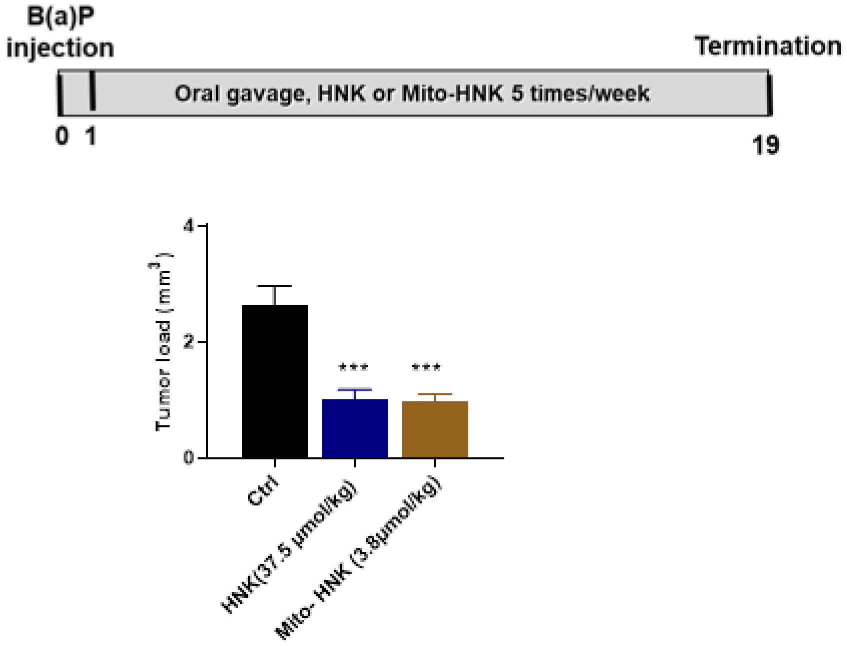

Figure S1. Efficacy of HNK and Mito-HNK in the B[*a*]P-induced lung cancer model. Upper panel: experimental design; Lower pane: Efficacy on tumor load. ***$p < 0.001$.
